# Stereotactic Radiosurgery as Part of Multimodal Treatment in a Bulky Leptomeningeal Recurrence of Breast Cancer

**DOI:** 10.7759/cureus.523

**Published:** 2016-03-08

**Authors:** Matthew H Bertke, Eric C Burton, Joseph N Shaughnessy

**Affiliations:** 1 Department of Radiation Oncology, University of Louisville; 2 Department of Neurology, University of Louisville

**Keywords:** stereotactic radiosurgery, leptomeningeal, breast cancer, reirradiation, intrathecal chemotherapy

## Abstract

Breast cancer metastatic to the brain and/or leptomeningeal spread of disease is a frequently encountered clinical situation, especially given the extended course of disease in these patients. Systemic therapies can often effectively prolong extracranial disease control, making effective strategies to control central nervous system-based disease even more critical. We present a case of bulky leptomeningeal relapse of breast cancer in the setting of prior whole brain radiation therapy. In order to treat the patient’s bulky disease and leptomeningeal spread while avoiding the potential toxicities of repeat whole brain radiation, the patient was treated with frameless stereotactic radiosurgery and intrathecal chemotherapy. This is the first report of this treatment approach for leptomeningeal relapse of breast cancer. The patient had an excellent response to treatment and durable intracranial control.

## Introduction

Brain metastases are a common manifestation of breast cancer, with 10–16% of patients eventually being diagnosed with metastatic disease to the brain [1]. Of these patients, as many as 20% will develop leptomeningeal disease either at the initial diagnosis of metastatic intracranial disease or after initial treatment of the brain with surgery, radiation, or both [2]. The treatment of leptomeningeal carcinomatosis, if pursued at all, has been palliative in nature. The treatment options typically include radiation therapy, either systemic or intrathecal chemotherapy, or a combination of these modalities. Radiation therapy for leptomeningeal disease is typically whole brain radiation therapy (WBRT) with additional treatment fields utilized as needed to encompass other areas of leptomeningeal spread along the neuraxis [3]. We present the case of a breast cancer patient with leptomeningeal recurrence of metastatic brain disease treated with stereotactic radiosurgery (SRS) and intrathecal chemotherapy in the setting of prior WBRT and multiple lines of systemic chemotherapy. To our knowledge this is the first report of SRS used with intrathecal chemotherapy described in the literature. The authors' institutional review board approved this study and waived informed patient consent. 

## Case presentation

We present the case of a 35-year-old female originally diagnosed with invasive ductal carcinoma of the right breast at the age of 31. At the time of diagnosis, she was found to have locoregional disease as well as multiple metastatic lesions in the liver, axial skeleton, and mediastinum. A biopsy showed the tumor to be (estrogen receptors/progesterone receptors) ER/PR positive, and HER2/neu was overamplified. Initial staging magnetic resonance imaging (MRI) of the brain was negative for metastatic intracranial disease. The patient received local palliative radiation therapy followed by eventual bilateral mastectomy after contralateral breast disease was discovered. She was treated with various systemic agents, including trastuzumab, docetaxel, lapatinib, and tamoxifen, before achieving a widespread response on eribulen.

Fourteen months after her initial diagnosis, the patient developed balance problems, and an MRI of the brain showed four enhancing nodules in the right cerebellum. The patient was treated with WBRT to a dose of 3,000 cGy in 10 fractions, and resolution of her intracranial disease was noted on subsequent brain MRI. The patient’s systemic disease was relatively stable over the next two and a half years with intervals of both progression and response to treatment and on various systemic therapies, including ado-trastuzumab emtansine and anastrazole.

**Figure 1:**
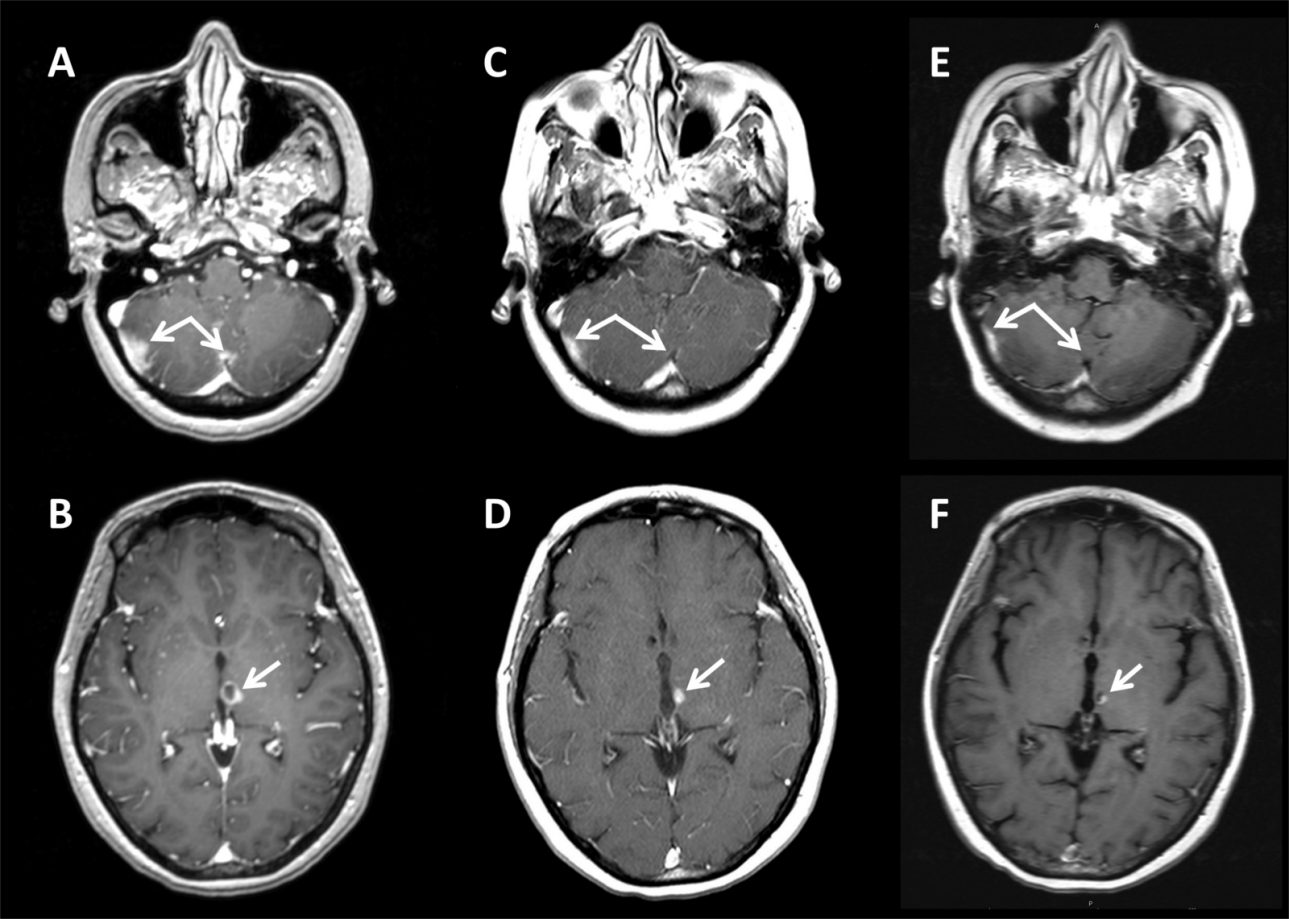
Brain MRI Findings Axial T1 post-contrast MRI shows areas of concern and response to treatment at two representative slices at the time of diagnosis of leptomeningeal relapse (A and B), one month post-radiosurgery (C and D), and five months post-radiosurgery (E and F). Dual arrows (A, C, E) highlight an area of bulky cerebellar disease and leptomeningeal involvement. Single arrows (B, D, F) highlight an area of bulky subventricular disease.

Repeat imaging of the brain performed 31 months after completion of her WBRT showed multiple new enhancing parenchymal and subventricular lesions and MRI findings consistent with leptomeningeal disease (Figures 1A-1B). Seven discrete bulky lesions were seen, with the largest lesion measuring 1.5 cm in the right cerebellum and the remainder measuring less than 1 cm. There was evidence of leptomeningeal involvement of the cerebellar fissures throughout the posterior fossa, and MRI of the complete neuraxis showed possible leptomeningeal caking in the patient’s lumbar nerve roots. At this time, the patient’s neurologic complaints consisted of mild ataxia and right occipital headaches, but she was free from any other focal deficits on neurologic examination. The patient’s case was discussed at a multidisciplinary neurooncology tumor board, with input from neuroradiology, neurooncology, radiation oncology, and neurosurgery. In light of her prior whole brain radiation therapy, the bulk of recurrent intracranial disease, and the presence of leptomeningeal carcinomatosis, a combined treatment approach using SRS followed by intrathecal chemotherapy was designed.

**Figure 2:**
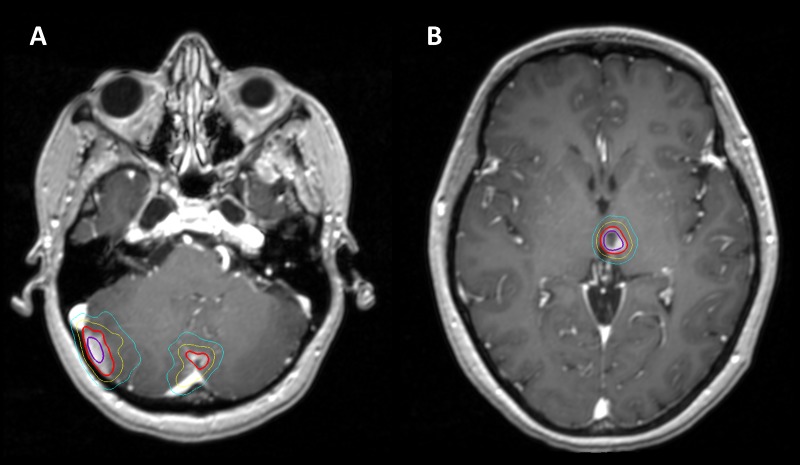
Stereotactic Radiosurgery Isodose Plan Representative axial depictions of the radiosurgery isodose plan for a site of cerebellar disease (A) and an area of bulky subventricular disease (B) are shown. Isodose curves are shown for 900 cGy (cyan), 1,200 cGy (yellow), 1,500 cGy (red), and 1,700 cGy (purple).

A course of SRS consisting of 1,500 cGy in a single fraction was delivered to all seven discrete intracranial lesions, including three right cerebellar lesions, a mid-cerebellar lesion, a left cerebellar lesion, a left thalamic lesion, and a left frontal lobe lesion. Radiation was delivered in a single unified plan using the frameless Cyberknife (Accuray Inc, Sunnyvale, CA) robotic linear accelerator-based treatment system. Motion management was achieved utilizing skull-tracking based on orthogonal imaging. This approach allowed the creation of a single complex plan to treat each discrete lesion using multiple isocenters (Figure 2). High conformality and rapid dose falloff was achieved by utilizing 287 discrete 6 MV photon pencil beamlets prescribed to the 78% isodose level. An Ommaya reservoir was placed and the patient was initiated on twice weekly intrathecal methotrexate (MTX). Each cycle consisted of 12 mg of MTX instilled with 3 ml of fluid into the Ommaya reservoir. After eight cycles of intrathecal chemotherapy and one month after the completion of SRS, a restaging MRI of the brain and neuraxis showed interval response of all previously noted leptomeningeal and parenchymal brain disease (Figures 1C-1D). At this time, the patient also had resolution of her previous right occipital headaches. Intrathecal MTX was continued for a total of nineteen cycles.

The patient continued to have a durable response intracranially without symptomatic neurologic complications or new neurologic exam findings for the next five months. Unfortunately the patient developed widespread hepatic and osseous metastatic disease and eventually declined further active systemic therapy. The patient died five months after completion of radiosurgery for leptomeningeal disease, with imaging consistent with controlled intracranial disease and without symptomatic neurologic decline (Figures 1E-1F).

## Discussion

This case represents a challenging but not uncommon clinical scenario. Leptomeningeal spread of malignancy is well described in breast cancer patients, occurring in up to 20% of those with brain metastases, either at initial diagnosis or after prior treatment [2]. The previous delivery of WBRT, although given 31 months prior, is a clinically relevant complicating factor that impacts future intracranial treatment approaches. The patient described in this case was symptomatic from her relapsed intracranial disease and desired aggressive treatment, despite her poor overall prognosis. The decision to treat using SRS and intrathecal chemotherapy was made for three main reasons.

First, intrathecal chemotherapy was considered a necessary component of treatment in order to address the widespread areas of leptomeningeal spread seen on imaging as well as at risk areas not grossly involved on imaging at the time of diagnosis of relapse.

Second, WBRT in the setting of intrathecal chemotherapy and prior WBRT was deemed to carry an excessive risk of toxicity. Repeat WBRT in the setting of prior WBRT carries an increased risk of adverse neurocognitive and neurologic treatment sequelae, and its efficacy is debated [4-5]. Additionally, the toxicity of WBRT is potentially amplified when given concurrently or adjacent to intrathecal chemotherapy [6]. Compared to WBRT, SRS has the benefit of exposing a smaller volume of normal brain to high doses of radiation. The clinical effects of combined SRS and intrathecal chemotherapy are not well described in the literature prior to this report, but limiting the area of brain exposed to both radiation and intrathecal chemotherapy has a theoretical benefit in reducing the likelihood of adverse treatment effects. This was thought to be especially true in the case described here, given the patient’s prior WBRT.

Lastly, the bulk of disease made the effectiveness of intrathecal chemotherapy alone less likely without the addition of radiation. Although intrathecal chemotherapy is ideally suited to deliver therapy across all surfaces exposed to cerebrospinal fluid, the depth of penetration in tumors can be as little as 1–2 mm [7]. For this reason, additional treatment with SRS was employed to adequately treat the areas of gross disease while diminishing the likelihood of adverse neurologic effects by minimizing the amount of normal brain receiving radiation. The overall result was a treatment approach utilizing SRS to manage the larger sites of disease and intrathecal chemotherapy to broadly treat areas of known and potential metastatic spread. This course of treatment had the desired effect of both palliating the patient’s headache in the short term as well as providing durable intracranial control of metastatic disease for the remainder of the patient’s life. Further neurologic adverse effects, from both the patient’s disease and subsequent treatment, were avoided.

Considering the poor prognosis associated with leptomeningeal disease, special care must be taken to appropriately select patients who may be eligible for aggressive treatment, such as what has been described above. Those with favorable histologies known to respond to systemic therapy, minimal extracranial disease burden, and/or good performance status may particularly benefit from such approaches. The addition of SRS is particularly fit for those who have received prior in-field radiation or have bulky disease that may not respond to lower doses of radiation.

## Conclusions

The presence of leptomeningeal disease is an extremely poor prognostic factor, with reported survival from several weeks up to four to six months depending on the choice of treatment [3]. Contrasting this with typical survival duration after the diagnosis of breast cancer metastatic to the brain, which is often a year or more, the direness of a diagnosis of leptomeningeal disease is apparent. Due to the poor outcomes seen with untreated leptomeningeal disease, it is reasonable to consider aggressive treatment approaches based on the patient’s goals of care. This is especially true when an important goal of treatment is avoiding neurologic death or compromise. The case described here demonstrates a potential treatment technique for patients who desire aggressive treatment for leptomeningeal metastases in the setting of prior WBRT.

## References

[1] Leone JP, Leone BA (2015). Breast cancer brain metastases: the last frontier. Exp Hematol Oncol.

[2] Vincent A, Lesser G, Brown D, Vern-Gross T, Metheny-Barlow L, Lawrence J, Chan M (2013). Prolonged regression of metastatic leptomeningeal breast cancer that has failed conventional therapy: a case report and review of the literature. J Breast Cancer.

[3] Feyer P, Sautter-Bihl ML, Budach W, Dunst J, Haase W, Harms W, Sedlmayer F, Souchon R, Wenz F, Sauer R (2010). DEGRO: practical guidelines for palliative radiotherapy of breast cancer patients: brain metastases and leptomeningeal carcinomatosis. Strahlenther Onkol.

[4] Mayer R, Sminia P (2008). Reirradiation tolerance of the human brain. Int J Radiat Oncol Biol Phys.

[5] Son CH, Jimenez R, Niemierko A, Loeffler JS, Oh KS, Shih HA (2012). Outcomes after whole brain reirradiation in patients with brain metastases. Int J Radiat Oncol Biol Phys.

[6] Calderoni A, Aebi S (2002). Combination chemotherapy with high-dose methotrexate and cytarabine with or without brain irradiation for primary central nervous system lymphomas. J Neurooncol.

[7] Huang T-Y, Arita N, Hayakawa T, Ushio Y (1999). ACNU, MTX and 5-FU penetration of rat brain tissue and tumors. J Neurooncol.

